# Solving a class of generalized fractional programming problems using the feasibility of linear programs

**DOI:** 10.1186/s13660-017-1420-1

**Published:** 2017-06-24

**Authors:** Peiping Shen, Tongli Zhang, Chunfeng Wang

**Affiliations:** College of Mathematics and Information Science, Henan Normal University, Xinxiang, 453007 P.R. China

**Keywords:** 90C30, 90C33, 90C15, generalized fractional programming, global optimization, approximation algorithm, computational complexity

## Abstract

This article presents a new approximation algorithm for globally solving a class of generalized fractional programming problems (P) whose objective functions are defined as an appropriate composition of ratios of affine functions. To solve this problem, the algorithm solves an equivalent optimization problem (Q) via an exploration of a suitably defined nonuniform grid. The main work of the algorithm involves checking the feasibility of linear programs associated with the interesting grid points. It is proved that the proposed algorithm is a fully polynomial time approximation scheme as the ratio terms are fixed in the objective function to problem (P), based on the computational complexity result. In contrast to existing results in literature, the algorithm does not require the assumptions on quasi-concavity or low-rank of the objective function to problem (P). Numerical results are given to illustrate the feasibility and effectiveness of the proposed algorithm.

## Introduction

In a variety of applications, we encounter a class of nonconvex optimization problems as follows:

$$ {\mathrm{(P)}:}\quad \textstyle\begin{cases} \min f(x)=G ( \frac{c_{1}^{\top}x+c_{01}}{d_{1}^{\top }x+d_{01}},\frac{c _{2}^{\top}x+c_{02}}{d_{2}^{\top}x+d_{02}}, \ldots,\frac{c_{p}^{ \top}x+c_{0p}}{d_{p}^{\top}x+d_{0p}} ) \\ \text{s.t.}\quad x\in\Omega=\{x\in\mathbb{R}^{n} : Ax\leq b,x\geq0\}, \end{cases} $$

where $c_{i}, d_{i}\in\mathbb{R}^{n}$, $c_{0i}, d_{0i}\in \mathbb{R}$, $A\in\mathbb{R}^{m\times n}$, $b\in\mathbb{R}^{m}$, $c_{i}^{\top}x+c_{0i}>0$, $d_{i}^{\top}x+d_{0i}>0$ over a nonempty, compact set Ω for each $i=1,\ldots,p$, and $G:\mathbb{R}_{+} ^{p}\rightarrow\mathbb{R}_{+}$ is a continuous function.

Problem (P) is worth studying because some important special optimization problems that have been studied in literature fall into the category of (P), such as multiplicative programs, sum-of-ratios optimization, fractional polynomial optimization, namely:

- *Multiplicative programs (MP)*: In this case, the objective function *G*, with the form $G(y_{1},\ldots,y_{p})=\prod_{i=1}^{p}y_{i}$ with $y_{i}=\frac{c_{i}^{\top}x+c_{0i}}{d_{i}^{ \top}x+d_{0i}}$, is quasi-concave, and its minimum is attained at some extreme point of the polytope [1]. Multiplicative objective functions arise in a variety of practical applications, such as economic analysis [2], robust optimization [3], VLSI chip design [4], combination optimization [5], etc.
- *Sum-of-ratios (SOR) optimization*: SOR functions have the form $G(y_{1},\ldots,y_{p}) =\sum_{i=1}^{p}y_{i}$ with $y_{i}=\frac{c_{i}^{\top}x+c_{0i}}{d_{i}^{\top}x+d_{0i}}$. Matsui [6] points out that it is NP-hard to minimize SOR functions over a polytope. For many applications of this form, we can see the survey paper by Schaible and Shi [7] and the references therein. Specially, a kind of SOR optimization problems with the form $G(y_{1},\ldots,y_{p})=\sum_{i=1}^{p} \vert y_{i} \vert ^{q}$, where $q\geq0$ and $y_{i}=\frac{c_{i}^{\top}x+c_{0i}}{d_{i}^{\top}x+d _{0i}}$, are considered by Kuno and Masaki [8] as well, and they often occur in a computer version.
- *Fractional polynomial optimization*: Polynomial functions with positive coefficients have the form $G(y_{1},\ldots,y _{p})=\sum_{j=1}^{m}c_{j}\prod_{i=1}^{p}y_{i}^{\gamma_{ij}}$, where $y_{i} =\frac{c_{i}^{\top}x+c_{0i}}{d_{i}^{\top}x+d_{0i}}$, $c_{j}\geq0$ and $\gamma_{ij}$ is a positive integer. Problems of this form have many applications [9], including production planning, engineering design, etc. In addition, from research point of view, these problems pose significant theoretical and computational challenges because they possess multiple local optima that are not globally optima.

During the past years, many solution methods have been developed for globally solving special cases of problem (P). These methods can be classified into outer-approximation [10], branch-and-bound [11–14], mixed branch-and-bound and outer-approximation [15], cutting plane [16], parameter-based [17], vertex enumeration [8], heuristic methods [18], etc. However, most of these methods lack theoretical analysis of the running time of the algorithms, or performance guarantee of the solutions obtained. To our knowledge, little work has been done about the solution of *ε*-approximation problems of (P) without the quasi-concavity and low-rank assumptions; although Locatelli [19] has developed an approximation algorithm for a general class of global optimization problems. Next, we immediately introduce the definition of the *ε*-approximation problem related to global optimization as follows.

### Definition 1

Given $\varepsilon>0$, letting $f_{\ast}=\min_{x\in\Omega}f(x)$, a point $\bar{x}\in \Omega$ is said to be an *ε*-approximation solution for $\min_{x\in\Omega}f(x)$ if

$$ f(\bar{x})\leq f_{\ast}+\varepsilon \vert f_{\ast} \vert . $$

This article focuses on presenting a fully polynomial time approximation scheme (FPTAS) for solving problem (P). An FPTAS for a minimization problem is an approximation algorithm, that is, for any given $\varepsilon>0$, it can find an *ε*-approximation solution for the problem, and its running time is polynomial in the input size of the problem and $1/\varepsilon$. As shown by Mittal and Schulz [20], the optimum value of problem (P) cannot be approximated to within any factor unless $\mathit{NP}=P$. Therefore, in order to obtain an FPTAS for solving problem (P), some extra assumptions of the function *G* will be required (see Section 2) in this article.

For the special cases of problem (P), many solution algorithms have been developed about the solution of *ε*-approximation problems. Depetrini and Locatelli [21] presented an approximation algorithm for linear fractional-multiplicative problems, and they pointed out that the algorithm is an FPTAS as the number *p* of ratio terms is fixed. This result has been extended to a wider class of optimization problems by Locatelli [19]. Also, Goyal and Ravi [22] exploited the fact that the minimum of a quasi-concave function is attained at an extreme point of the polytope and proposed an FPTAS for minimizing a class of low-rank quasi-concave functions over a convex set. Mittal and Schulz [20] developed an FPTAS for optimizing a class of low-rank nonconvex functions without quasi-concavity over a polytope. In addition, Depetrini et al. [23] and Goyal et al. [24] respectively gave an FPTAS for a class of optimization problems where the objective functions are products of two linear functions. Shen and Wang [25] presented a linear decomposition approximation algorithm for a class of nonconvex programming problems by dividing the input space into polynomially many grids. Nevertheless, these solution methods [20, 21, 23–25] cannot be directly applied to the case (i.e., problem (P)) considered in this paper, where the objective function is a composition of some ratios of affine functions without quasi-concavity or low-rank.

The aim of this article is to present a solution approach for a class of fractional programming problems (P). By introducing some variables, the original problem (P) is first converted to a *p*-dimensional equivalent problem (Q). Through the establishment of a nonuniform grid, on the basis of problem (Q), the solving process of the original problem (P) is then transformed into checking the feasibility of a series of linear programming problems. Thus, a new approximation algorithm is presented for globally solving problem (P) based on the exploration technique of a nonuniform grid over a box. The algorithm does not require quasi-concavity or low-rank of the function *G* to problem (P), and it is proved that this is an FPTAS as the term *p* is fixed in *G*. We emphasize here that the exploration technique used in this article is different from the ones given in [19, 21]. The reason is that we utilize a different strategy from that given in [19, 21] to update the incumbent best value of the objective function $g(t)$ to problem (Q), and that requires fewer interesting grid points restored and considered in our algorithm, compared with Refs. [19, 21]. Also, we notice that the main computational cost of the proposed algorithm is checking the feasibility of linear problems at the interesting grid points. This means that it requires less computational cost and so is more easily implementable. Finally, problem (P) generalizes the one investigated in [21], and the proposed algorithm can be directly applied to solve the problem in [19] by replacing the convex feasibility with the linear one. Numerical results show that the proposed algorithm requires much less computational time to obtain an approximation optimized solution of problem (P) with the same approximation error than the approaches (given by [19, 21]) do.

The paper is structured as follows. In Section 2, we discuss the reformulation of problem (P) as a *p*-dimensional one. Section 3 presents an approximation algorithm to obtain an *ε*-approximation solution for problem (P) which is FPTAS by its computational complexity. Some numerical results are reported in Section 4. Finally, the conclusions are presented in Section 5.

## Parametric reformulation of the problem

For solving problem (P), throughout this paper, we assume that *G* satisfies:

- $G(y)\leq G(y^{\prime})$ for all $y, y^{\prime}\in\mathbb{R}_{+} ^{p}$ with $y_{i}\leq y_{i}^{\prime}$, $i=1,\ldots,p$, and
- $\delta^{k}G(y)\leq G(\delta y)$ for all $y\in\mathbb{R}_{+}^{p}$, $\forall\delta\in(0,1)$, and some constant *k*.

There are a number of functions *G* which satisfy the above conditions, such as the product of a constant number (say *p*) of linear functions (with $k=p$), the sum of linear ratio functions (with $k=1$), etc. This paper will present an approximation algorithm for solving problem (P) under the above assumptions. For this purpose, let us introduce *p* variables $y_{i}$, $i=1,\ldots,p$, thus, problem (P) can be equivalent to the form:

$$ {\mathrm{(P1)}:}\quad \textstyle\begin{cases} \min G(y) \\ \text{s.t.}\quad \frac{c_{i}^{\top}x+c_{0i}}{d_{i}^{\top}x+d_{0i}}\leq y _{i}, \quad i=1,\ldots,p, \\ \hphantom{\text{s.t.}\quad}x\in\Omega. \end{cases} $$

### Theorem 1

$x^{\ast}$ *is a global optimal solution for problem* (P) *if and only if* $(x^{\ast},y^{\ast})$ *is a global optimal solution for problem* (P1) *with* $y^{\ast}_{i}=\frac{c_{i}^{\top}x^{\ast}+c_{0i}}{d _{i}^{\top}x^{\ast}+d_{0i}}$ *for each* $i=1,\ldots,p$. *The minimal objective function values of problems* (P) *and* (P1) *are equal*, *i*.*e*., $f(x^{\ast})=G(y^{\ast})$.

### Proof

Let $(x^{\ast},y^{\ast})$ be a global optimal solution for problem (P1). We suppose that $x^{\ast}$ is not a global optimal solution for problem (P), then there exists $\bar{x}\in\Omega$ such that

2.1 $$ f(\bar{x})< f\bigl(x^{\ast}\bigr). $$

Let $\bar{y}_{i}=\frac{c_{i}^{\top}\bar{x}+c_{0i}}{d_{i}^{\top} \bar{x}+d_{0i}}$, $i=1,\ldots,p$. Then $(\bar{x},\bar{y})$ is a feasible solution of problem (P1). We can have, from (2.1), that

2.2 $$ G(\bar{y})=f(\bar{x})< f\bigl(x^{\ast}\bigr). $$

On the other hand, since $(x^{\ast},y^{\ast})$ is a feasible solution of problem (P1), this implies that $\frac{c_{i}^{\top}x^{\ast}+c _{0i}}{d_{i}^{\top}x^{\ast}+d_{0i}}\leq y^{\ast}_{i}$, $i=1,\ldots,p$. Therefore, from the assumptions of *G*, it holds that

2.3 $$ f\bigl(x^{\ast}\bigr)=G \biggl( \frac{c_{1}^{\top}x^{\ast}+c_{01}}{d_{1}^{ \top}x^{\ast}+d_{01}}, \frac{c_{2}^{\top}x^{\ast}+c_{02}}{d_{2} ^{\top}x^{\ast}+d_{02}}, \ldots,\frac{c_{p}^{\top}x^{\ast}+c_{0p}}{d _{p}^{\top}x^{\ast}+d_{0p}} \biggr) \leq G\bigl(y^{\ast} \bigr). $$

Combining (2.2) with (2.3), we can obtain $G(\bar{y})< G(y ^{\ast})$. Since $(\bar{x},\bar{y})$ is a feasible solution of problem (P1), this contradicts the optimality of $(x^{\ast},y^{\ast})$ for problem (P1). Therefore, the supposition that $x^{\ast}$ is not a global optimal solution for problem (P) must be false.

Next, we will show the converse case. Let $x^{\ast}$ be a global optimal solution of problem (P), and let $y^{\ast}_{i}=\frac{c_{i} ^{\top}x^{\ast}+c_{0i}}{d_{i}^{\top}x^{\ast}+d_{0i}}$, $i=1,\ldots,p$. Then $(x^{\ast},y^{\ast})$ is a feasible solution of problem (P1). Suppose that there exists some feasible solution $(\bar{x},\bar{y})$ for problem (P1) such that

2.4 $$ G(\bar{y})< G\bigl(y^{\ast}\bigr)=f\bigl(x^{\ast} \bigr). $$

Then, from $\frac{c_{i}^{\top}\bar{x}+c_{0i}}{d_{i}^{\top}\bar{x}+d _{0i}}\leq\bar{y}_{i}$, $i=1,\ldots,p$, it follows that

2.5 $$ f(\bar{x})=G \biggl( \frac{c_{1}^{\top}\bar{x}+c_{01}}{d_{1}^{\top} \bar{x}+d_{01}},\frac{c_{2}^{\top}\bar{x}+c_{02}}{d_{2}^{\top} \bar{x}+d_{02}}, \ldots,\frac{c_{p}^{\top}\bar{x}+c_{0p}}{d_{p}^{ \top}\bar{x}+d_{0p}} \biggr) \leq G(\bar{y}). $$

By using (2.4)-(2.5), we have $f(\bar{x})< G(y^{\ast})=f(x ^{\ast})$. Since $\bar{x}\in\Omega$, this contradicts that $x^{\ast}$ is an optimal solution of problem (P). Hence, $(x^{\ast},y ^{\ast})$ must be the optimal solution to (P1). Based on the above result, obviously, from the assumptions of *G*, we have $f(x^{\ast})=G(y ^{\ast})$. □

Based on the above theorem, for solving problem (P), we may solve problem (P1) instead. Additionally, it is known that each single ratio $\frac{c_{i}^{\top}x+c_{0i}}{d_{i}^{\top}x+d_{0i}}$ is both quasi-concave and quasi-convex, and its minimum and maximum must be attained respectively at some vertex of Ω (see, e.g., [26]). To this end, let us denote

2.6 $$ l_{i}=\min_{x\in\Omega}\frac{c_{i}^{\top}x+c_{0i}}{d_{i} ^{\top}x+d_{0i}},\quad\quad u_{i}=\max_{x\in\Omega}\frac{c_{i}^{\top}x+c_{0i}}{d_{i} ^{\top}x+d_{0i}}, \quad i=1,\ldots,p. $$

And let

$$ H=\bigl\{ y\in\mathbb{R}^{p} : l_{i}\leq y_{i} \leq u_{i}, i=1,\ldots,p \bigr\} . $$

Now, let us define a *p*-dimensional set for each $t\in H$ as follows:

$$ S(t)=\bigl\{ x\in\Omega: c_{i}^{\top}x+c_{0i}\leq t_{i}\bigl(d_{i}^{\top}x+d _{0i}\bigr), i=1,\ldots,p\bigr\} , $$

and the corresponding function $g(t)$ is given by

$$ g(t) = \textstyle\begin{cases} G(t), & \textrm{if } S(t)\neq\emptyset , \\ +\infty,& \textrm{otherwise.} \end{cases} $$

Clearly, we can know whether $S(t)$ is a null set or not by checking the feasibility of a linear program for given $t\in H$, which can be solved in polynomial time. Based on the above result, it turns out that problem (P1) is equivalent to the following *p*-dimensional problem:

$$ {\mathrm{(Q)}:} \quad \min_{t\in H} g(t). $$

According to the definition of $g(t)$, we have the following conclusion.

### Theorem 2

*Given* $\varepsilon>0$, *let* $\delta=(\frac{1}{1+\varepsilon})^{1/k}$, *for each* $\bar{t}\in H$, *it holds that*

$$ g(\bar{t})\leq(1+\varepsilon)g(t), \quad \forall t\in[\delta\bar{t}, \bar{t} ]. $$

### Proof

From the definition of $S(t)$ and $\delta=(\frac{1}{1+\varepsilon})^{1/k} \in(0,1)$, we have $S(\delta\bar{t})\subseteq S(\bar{t})$ for each $\bar{t}\in H$. When $S(\delta\bar{t})\neq\emptyset$, it implies that $S(t)\neq\emptyset$ for each $t\in[\delta\bar{t},\bar{t}]$. This means that $g(t)=G(t)$ for each $t\in[\delta\bar{t},\bar{t} ]$. With the assumptions of $G(t)$, it holds that

$$ (1+\varepsilon)g(t)=(1+\varepsilon)G(t)\geq(1+\varepsilon)G( \delta\bar{t}) \geq(1+\varepsilon)\delta^{k}G(\bar{t})=G(\bar{t})=g( \bar{t}), \quad \forall t\in[\delta\bar{t},\bar{t} ]. $$

When $S(\delta\bar{t})=\emptyset$ and $S(\bar{t})\neq\emptyset$, similarly, we have that

$$ (1+\varepsilon)g(t)\geq(1+\varepsilon)G(t)\geq(1+\varepsilon)G( \delta\bar{t}) \geq(1+\varepsilon)\delta^{k}G(\bar{t})=G(\bar{t})=g( \bar{t}),\quad \forall t\in[\delta\bar{t},\bar{t} ]. $$

When $S(\bar{t})=\emptyset$, it implies that $S(t)=\emptyset$ for any $t\in[\delta\bar{t},\bar{t}]$, and so the conclusion holds. □

## The approximation algorithm

### The algorithm and its convergence

In this subsection, by using Theorem 2 above, we present an approximation algorithm for solving problem (P), and prove that the algorithm can find an *ε*-approximation solution for problem (P).

The proposed algorithm adopts an exploration technique of a suitably defined nonuniform grid over *H*. In the algorithm, let $\mathcal{T}$ be the set of all restored interesting grid points which will be further analyzed. $\mathcal{W}$ is a set of the grid points already discarded, and $\mathcal{X}$ is a set of the remaining grid points at each iteration. Moreover, *U* represents the best value of the function $g(t)$ obtained so far, and denote $t^{\ast}$ such that $U=g(t^{ \ast})$. The algorithm starts with $t^{\ast}=(u_{1},\ldots,u_{p})$ and $U=g(t^{\ast})$. In each iteration, we select a point $\bar{t} \in\mathcal{T}$ and calculate $\bar{a}=\min\{a\in\mathbb{N}: S( \delta^{a}\bar{t} )=\emptyset\}$, where $\mathbb{N}$ represents the set of the natural numbers. If $\bar{a}=0$, we newly select a point *t̄* from $\mathcal{T}$. Otherwise, we have $S(\delta^{\bar{a}-1} \bar{t})\neq\emptyset$, and so $S(t)\neq\emptyset$ for each $t\in[\delta^{\bar{a}-1}\bar{t},\bar{t}]$. This implies that $g(t)=G(t)$ for each $t\in[\delta^{\bar{a}-1}\bar{t},\bar{t}]$. By using the nondecreasing *G*, it holds that $g(\delta^{\bar{a}-1} \bar{t})=\min_{t\in[\delta^{\bar{a}-1}\bar{t},\bar{t}]}g(t)$. In addition, for any $t\in\{t:\delta^{\bar{a}}\bar{t}_{i}< t_{i} \leq\bar{t}_{i}, i=1,\ldots,p\}\triangleq(\delta^{\bar{a}}\bar{t}, \bar{t}]$, there exists an integer vector $\tau=(\tau_{1},\ldots, \tau_{p})$ such that $t_{i}\in(\delta^{\tau_{i}+1}\bar{t}_{i}, \delta^{\tau_{i}}\bar{t}_{i}]$ satisfying $\tau_{i}\in\{0,1,\ldots, \bar{a}-1\}$ for each *i*, thus, we have $(1+\varepsilon)g(t)\geq g( \delta^{\tau}\bar{t})$ for any $t\in(\delta^{\tau+1}\bar{t}, \delta^{\tau}\bar{t}]$ from Theorem 2. We see that all points $\delta^{\tau}\bar{t}=(\delta^{\tau_{1}}\bar{t}_{1},\ldots, \delta^{\tau_{p}}\bar{t}_{p})$ with $\tau_{i}\in\{0,1,\ldots, \bar{a}-1\}$ belong to $[\delta^{\bar{a}-1}\bar{t},\bar{t}]$, hence,

$$ \begin{aligned} (1+\varepsilon)g(t)&\geq\min_{\tau_{i}\in\{0,1,\ldots,\bar{a}-1\},\forall i} g\bigl( \delta^{\tau_{1}}\bar{t}_{1},\ldots,\delta^{\tau_{p}}\bar {t}_{p}\bigr)= g\bigl( \delta^{\bar{a}-1}\bar{t}\bigr) \\ &\geq\min\bigl\{ U,g\bigl(\delta^{\bar{a}-1}\bar{t}\bigr) \bigr\} , \quad \forall t\in( \delta^{\bar{a}}\bar{t},\bar{t} ]. \end{aligned} $$

And so, it is reasonable to update $U=\min\{U,g(\delta^{\bar{a}-1} \bar{t})\}$ and $t^{*}$ such that $g(t^{\ast})=U$. Next, we consider $2^{p}$ new points $(\xi_{1}\bar{t_{1}},\ldots,\xi_{p}\bar{t_{p}})$ with $\xi_{i}\in\{\delta^{\bar{a}},1\}$ for all *i*, discard all points which satisfy $\xi_{i}u_{i}< l_{i}$ for some *i*, and add the remaining points to $\mathcal{X}$, then update $\mathcal{T}=(\mathcal{T}\cup \mathcal{X})\backslash\mathcal{W}$. This process is repeated until $\mathcal{T}=\emptyset$. At termination, each point $x^{\ast}\in S(t ^{\ast})$ is an approximation solution of problem (P). The detailed algorithm is summarized as Algorithm 1.

**Algorithm 1:**
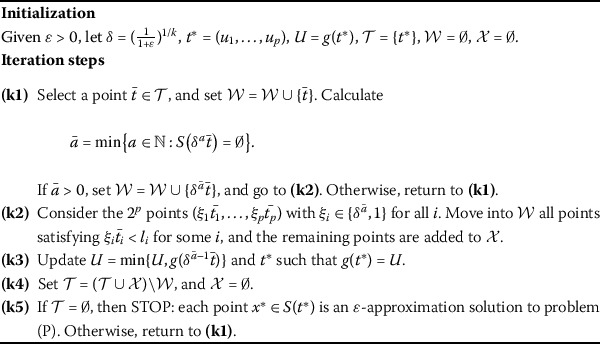
Approximation algorithm statement

#### Theorem 3

*The proposed algorithm can find an* *ε*-*approximation solution for problem *(P).

#### Proof

Note that the algorithm evaluates the function $g(t)$ values at the following points:

$$ \bigl(\delta^{s_{1}}u_{1},\ldots,\delta^{s_{p}}u_{p} \bigr), $$

where $s_{i}\in\mathbb{N}$, and satisfies

3.1 $$ 0\leq s_{i}\leq\bar{s_{i}}\triangleq\max \bigl\{ s : \delta^{s}u_{i} \geq l_{i}\bigr\} , \quad i=1,\ldots,p. $$

For any $t\in H$, there is an integer vector $(s_{1},\ldots,s_{p})$ with $0\leq s_{i}\leq\bar{s_{i}}$, $i=1,\ldots,p$, such that $t\in\prod_{i=1}^{p}[\delta^{s_{i}+1}u_{i},\delta^{s_{i}}u_{i}]$. Thus, in view of Theorem 2 and the definition of *δ*, it holds that $g(\delta^{s_{1}}u_{1},\ldots,\delta^{s_{p}}u_{p})\leq (1+\varepsilon )g(t)$ for each $t\in\prod_{i=1}^{p}[\delta^{s_{i}+1}u_{i},\delta ^{s_{i}}u_{i}]$. Hence, we have

$$ \min_{s_{i}\in\{0,1,\ldots,\bar{s_{i}}\},\forall i}g\bigl( \delta^{s_{1}}u_{1}, \ldots,\delta^{s_{p}}u_{p}\bigr)\leq(1+\varepsilon) \min _{t\in H}g(t). $$

On the other hand, let us denote $t^{*}=(\delta^{s_{1}^{*}}u_{1}, \ldots,\delta^{s_{p}^{*}}u_{p})$ such that

$$ g\bigl(t^{*}\bigr)=\min_{s_{i}\in\{0,1,\ldots,\bar{s_{i}}\},\forall i}g\bigl( \delta^{s_{1}}u_{1},\ldots,\delta^{s_{p}}u_{p} \bigr). $$

From Step ($k_{2}$) of the algorithm, we know $S(t^{*})\neq\emptyset$. By using the definition of $S(t)$, there exists a point $x^{*}$ satisfying $x^{*}\in S(t^{*})$. Now, let us denote $\tilde{t}_{i}=\frac{c_{i} ^{\top}x^{*}+c_{0i}}{d_{i}^{\top}x^{*}+d_{0i}}$, $i=1,\ldots,p$, then we have $x^{*}\in S(\tilde{t})$ and $\tilde{t}_{i}\leq t^{*}_{i}$. Combining the definition of $g(t)$, we see that $g(\tilde{t})\leq g(t ^{*})$. Thus, we conclude that

$$ (1+\varepsilon)\min_{x\in\Omega}f(x)=(1+\varepsilon) \min _{t\in H}g(t)\geq\min_{s_{i}\in\{0,1,\ldots,\bar{s_{i}}\},\forall i}g\bigl( \delta^{s _{1}}u_{1},\ldots,\delta^{s_{p}}u_{p} \bigr)=g\bigl(t^{*}\bigr)\geq g(\tilde{t})=f\bigl(x ^{*} \bigr). $$

Therefore, the point $x^{\ast}$ is an *ε*-approximation solution of problem (P) by Definition 1. □

### The complexity of the algorithm

In this subsection, the computational complexity of the algorithm will be presented in order to show that the approximation algorithm is an FPTAS for fixed *p*. For this purpose, we need to use the following lemma from Ref [27]. Let $\Omega=\{x\in\mathbb{R}^{n}: Ax\leq b,x\geq0\}$ be a polyhedron with $A\in\mathbb{R}^{m\times n}$, $b\in\mathbb{R}^{m}$, and denote

$$ \bar{\lambda}=\max\bigl\{ 1, \vert A_{ij} \vert , \vert b_{i} \vert : i=1,\ldots,m, j=1,\ldots ,n\bigr\} . $$

Then we have the following lemma.

#### Lemma 1

[27]

*Let* $x^{0}$ *be a vertex of* Ω, *then*, *for each* $j=1,\ldots,n$, *it holds that*

$$ x_{j}^{0}=p_{j}/q, $$

*where* $p_{j}\in\mathbb{R}$, $q\in\mathbb{R}$ *with*

3.2 $$ 0\leq p_{j}\leq(n\bar{\lambda})^{n}, \quad \quad 0< q\leq(n\bar{\lambda})^{n}. $$

#### Lemma 2

*Given* $\varepsilon>0$, *let* $\delta=(\frac{1}{1+\varepsilon})^{1/k}$. *The number of the points* $(\delta^{s_{1}}u_{1},\ldots,\delta^{s_{p}}u _{p})$ *satisfying* (3.1), *at which the feasibility of the corresponding linear programs are checked by the proposed algorithm*, *is not more than*

$$ \prod_{i=1}^{p} \biggl[ 1+ \frac{k}{\varepsilon}\ln\biggl(\frac{u_{i}}{l _{i}}\biggr) \biggr] . $$

#### Proof

Note that $\delta=(\frac{1}{1+\varepsilon})^{1/k}\in(0,1)$ is fixed if $\epsilon> 0$ is given. Since the points $(\delta^{s_{1}}u_{1}, \ldots, \delta^{s_{p}}u_{p})$ belonging to the nonuniform grid over *H* satisfy (3.1), the number of these grid points is equal to $\prod_{i=1}^{p}(\bar{s_{i}}+1)$. Moreover, by the proposed algorithm, the number of the points $(\delta^{s_{1}}u_{1},\ldots,\delta^{s_{p}}u _{p})$ at which the feasibility of linear programs should be checked is not larger than $\prod_{i=1}^{p}(\bar{s_{i}}+1)$. In view of the definition of $\bar{s_{i}}$ and *δ*, we can have that

$$ \bar{s_{i}}\leq\ln(l_{i}/u_{i})/(\ln\delta)= \bigl[k\ln(l_{i}/u_{i})\bigr]/ \ln(1+\varepsilon), \quad i=1, \ldots,p. $$

Since $\ln(1+\varepsilon)\approx\varepsilon$ for sufficiently small $\varepsilon>0$, we see that the number of points where the feasibility of linear programs should be checked is not larger than $\prod_{i=1} ^{p}[1+\frac{k}{\varepsilon}\ln(\frac{u_{i}}{l_{i}})]$. □

By the proposed algorithm, to find an *ε*-approximation solution for problem (P), the computational cost includes the cost of the computation of the box *H* and the calculation of *ā* at Step (k1) of the algorithm for each iteration. It is known that each $l_{i}$ and $u_{i}$ must be attained at some vertex of Ω respectively (see, e.g., [26]), and that can be computed in polynomial time, thus *H* can be determined in polynomial time. On the other hand, we notice that the main work is the calculation of *ā* at each iteration in the algorithm (see Step (k1)). This is because the calculation of *ā* at each iteration requires checking the feasibility of some linear problems with $m+p$ constraints and *n* variables. In other words, the computational cost of the algorithm is to check the feasibility of linear problems at interesting grid points. Let us denote $T(m+p,n)$ as the cost of checking the feasibility of a linear programming problem with $m+p$ constraints and *n* variables.

In order to give the computational cost of the proposed algorithm, without loss of generality, we can assume that

3.3 $$ c_{i}^{\top}x+c_{0i}\geq1, \quad\quad d_{i}^{\top}x+d_{0i}\geq1, \quad \forall x\in\Omega. $$

This is because

$$ \frac{c_{i}^{\top}x+c_{0i}}{d_{i}^{\top}x+d_{0i}}=\frac{M_{i}(c_{i} ^{\top}x+c_{0i})}{M_{i}(d_{i}^{\top}x+d_{0i})} , \quad i=1,\ldots,p, $$

by choosing sufficiently large $M_{i}\in\mathbb{R}$ such that $M_{i}(c_{i}^{\top}x+c_{0i})\geq1$, $M_{i}(d_{i}^{\top}x+d_{0i}) \geq1$ for any $x\in\Omega$. Based on the above discussion, combining Lemmas 1 and 2 finally leads to the following theorem.

#### Theorem 4

*As* *p* *is a fixed positive integer*, *the number of operations required by the proposed algorithm to obtain an* *ε*-*approximate solution for problem* (P) *is not larger than*

$$ O \biggl( \biggl[ \frac{2k(n+1)\ln(n\lambda)}{\varepsilon} \biggr] ^{p} T(m+p,n) \biggr) , $$

*where* $\lambda=\max\{\bar{\lambda}, \vert c_{ij} \vert , \vert d_{ij} \vert , \vert c_{0i} \vert , \vert d_{0i} \vert : i=1,\ldots,p, j=1,\ldots,n\}$.

#### Proof

Let $x^{l_{i}}$, $x^{u_{i}}$ be vertices of Ω with $l_{i}=\frac{c _{i}^{\top}x^{l_{i}}+c_{0i}}{d_{i}^{\top}x^{l_{i}}+d_{0i}}$, $u_{i}=\frac{c_{i}^{\top}x^{u_{i}}+c_{0i}}{d_{i}^{\top}x^{u_{i}}+d _{0i}}$, $i=1,2,\ldots,p$. Thus, it follows from Lemma 1 that

$$ x_{j}^{l_{i}}=p_{j}^{l_{i}}/q^{l_{i}},\quad\quad x_{j}^{u_{i}}=p_{j}^{u_{i}}/q ^{u_{i}}, \quad j=1,\ldots,n, i=1,\ldots,p, $$

where $p_{j}^{l_{i}}$, $q^{l_{i}}$, $p_{j}^{u_{i}}$, $q^{u_{i}}$ satisfy (3.2). Let $\rho=\max\{1, 1/q^{l_{i}},1/q^{u_{i}}: i=1,\ldots ,p\}$. Combining Lemma 1 and the definition of *λ* leads to

$$ d_{i}^{\top}x^{l_{i}}+d_{0i}=\sum _{j=1}^{n}d_{ij}p_{j}^{l_{i}}/q ^{l_{i}}+d_{0i}\leq\rho\sum_{j=1}^{n}d_{ij}p_{j}^{l_{i}}+ \lambda\leq\rho n^{n+1}\lambda^{n+1}+\lambda\leq2\rho n^{n+1} \lambda^{n+1}. $$

Thus, with (3.3), it holds that

$$ l_{i}=\bigl(c_{i}^{\top}x^{l_{i}}+c_{0i} \bigr)/\bigl(d_{i}^{\top}x^{l_{i}}+d_{0i}\bigr) \geq1/\bigl(2\rho n^{n+1}\lambda^{n+1}\bigr). $$

Similarly, we can obtain that $u_{i}\leq2\rho n^{n+1}\lambda^{n+1}$. And so

$$ \ln(u_{i}/l_{i})\leq\ln\bigl(4\rho^{2}n^{2n+2} \lambda^{2n+2}\bigr)=2\ln(2 \rho)+2(n+1)\ln(n\lambda). $$

Since for each interesting grid point we require the solution of a linear feasibility problem with $m+p$ constraints and *n* variables, by Lemma 2, for given *p*, we can claim that the number of operations required by the proposed algorithm is not larger than

$$ \begin{gathered} \biggl[ 1+\frac{2 k\ln(2\rho)+2k(n+1)\ln(n\lambda)}{\varepsilon } \biggr] ^{p}T(m+p,n) \\ \quad =O \biggl( \biggl[ \frac{2k(n+1)\ln(n\lambda )}{\varepsilon} \biggr] ^{p} T(m+p,n) \biggr) . \end{gathered} $$

 □

#### Remark 1

From Theorem 4, we can conclude that the proposed algorithm is an FPTAS for problem (P) for fixed *p*. On the other hand, we know that the computational time of the proposed algorithm is an exponential increase with *p* increasing. These conclusions can be observed also in the numerical results of the next section.

#### Remark 2

Notice that the detailed complexity analysis of the proposed algorithm can be used as an indicator of the difficulty of some optimization problems, such as multiplicative programs, sum-of-ratios optimization, etc. Thus, in order to solve efficiently these problems, we should expect to design a more sophisticated approach where its performance is at least as good.

## Numerical examples

Based on Theorem 4, although the computational complexity results of the algorithms ([19, 21] and ours) are similar, we should notice that it is the worst case time complexity which is one of the most often used criteria of evaluating algorithms in optimization. In fact, these complexity results ([19, 21] and ours) are only the upper bounds of the computational cost of the algorithms for solving optimization problems under the worst case, i.e., all the grid points are considered. Hence, to further verify the performance of the proposed algorithm in this article, in this section we compare the proposed algorithm with the ones in [19, 21] by numerical examples. Because it is an approximation algorithm for solving general fractional programming problem (P), we do not attempt comparisons with the solution methods for solving special cases of (P) (e.g., branch-and-bound [11, 12], outer-approximation [15], cutting plane [16], etc.), and the approximation algorithms in [20, 22], which are restricted to solving problems under the quasi-concavity or low-rank assumptions in the objective functions. Additionally, the algorithms ([19, 21] and ours) are based on the exploration of a suitably defined nonuniform grid over a rectangle, but we exploit different exploration strategies to minimize the objective function over the feasible set, and use different methods to update the incumbent best value of the objective function obtained at each iteration, compared with [19, 21].

We implemented the three algorithms ([19, 21] and ours) in MATLAB 2012b with some test experiments. Tests are run on a PC with dual processor CPU (2.33 Hz), Intel(R), and Core(TM) i3. Notice that these algorithms use different approaches for computing the lower bound $l_{i}$ and the upper bound $u_{i}$ of each ratio term in the objective functions. Hence, for comparison, each $l_{i}$, $u_{i}$ in the three algorithms is given by taking the same way (i.e., using (2.6)) in our computation.

Some notations in Tables 1, 2, 3 have been used for column headers: Solution: the approximate optimal solution; Optimum: the approximate optimal value; Iter: the number of the algorithm iterations; CPU(s): the execution time in seconds; Nodes: the maximal number of the interesting grid points restored; Avg: average performance by the algorithm; Std: standard deviation of performances by the algorithm.

**Table 1:** **Computational results of Examples** **1** **-** **5**

	**Algorithm**	***ε***	**Solution**	**Optimum**	**Iter**	**Nodes**	**CPU(s)**
1	[21]	0.2	(0, 0.2816)	1.6232	5,122	862	185.2
	[19]	0.2	(0, 0.2816)	1.6232	1,122	327	84.9
	Our	0.2	(0, 0.2816)	1.6232	17	5	0.46
2	[21]	0.2	(5.382 × 10^−16^, 5.536 × 10^−16^)	0.5333	631	217	10.4
	[19]	0.2	(5.382 × 10^−16^, 5.536 × 10^−16^)	0.5333	362	122	5.63
	Our	0.2	(5.382 × 10^−16^, 5.536 × 10^−16^)	0.5333	55	13	1.83
3	[21]	0.15	(0, 0, 1.6886, 4.3466, 4.3007, 4.0334, 0, 1.4324, 0.7765, 4.1967, 0, 4.1385)	0.05115	24,569	3,727	355.2
	[19]	0.15	(0, 0, 1.6886, 4.3466, 4.3007, 4.0334, 0, 1.4324, 0.7765, 4.1967, 0, 4.1385)	0.05115	14,669	3,215	215.2
	Our	0.15	(0, 0, 1.6886, 4.3466, 4.3007, 4.0334, 0, 1.4324, 0.7765, 4.1967, 0, 4.1385)	0.05115	70	21	5.66
4	[19]	0.1	(1.7177, 2.0155)	32.39	1,998	487	118.8
	Our	0.1	(1.7177, 2.0155)	32.39	41	15	32.39
5	[19]	0.05	(2.0814, 2.9963)	7,709.8	4,383	1,327	256.9
	Our	0.05	(2.0814, 2.9963)	7,709.8	924	385	56.2

**Table 2:** **Computational results of randomly generated test problems with** $\pmb{(m,n)=(50,50)}$

***p***	**Algorithm**	**CPU(s)**		**Iter**		**Nodes**	
		**Avg**	**Std**	**Avg**	**Std**	**Avg**	**Std**
2	[21]	46.5	24.1	1,369.4	75.3	362.6	35.9
	[19]	39.6	15.5	1,225.2	62.8	302.0	23.4
	Our	1.2	0.5	7.8	1.6	2.2	0.5
4	[21]	5,862.1	903.8	16,973.0	994.8	3,612.5	917.2
	[19]	4,590.7	802.5	17,888.1	913.8	3,294	534.1
	Our	206.2	70.8	3,144.4	172.1	912.8	111.8
5	[21]	7,102.2	913.8	29,121.3	904.8	9,612.5	982.5
	[19]	5,062.4	893.1	19,373.4	924.1	6,613.9	861.4
	Our	813.6	113.4	5,082.9	823.7	1,403.6	813.9
6	[21]	-	-	-	-	-	-
	[19]	6,384.7	895.2	38,359.4	921.7	11,869.8	938.2
	Our	1,455.7	201.2	9,830.4	485.7	2,769.3	216.6
7	[21]	-	-	-	-	-	-
	[19]	-	-	-	-	-	-
	Our	2,754.3	430.7	12,054.4	523.5	3,257.9	433.7
8	[21]	-	-	-	-	-	-
	[19]	-	-	-	-	-	-
	Our	4,175.6	603.2	19,853.4	873.3	5,107.7	513.2
9	[21]	-	-	-	-	-	-
	[19]	-	-	-	-	-	-
	Our	6,175.1	837.9	28,251.5	869.2	8,632.2	752.3
10	[21]	-	-	-	-	-	-
	[19]	-	-	-	-	-	-
	Our	7,075.9	997.8	33,215.7	963.8	9,897.4	924.3

**Table 3:** **Computational results of randomly generated test problems with** $\pmb{p=4}$

**[** ***m*** **,** ***n*** **]**	**Algorithm**	**CPU(s)**		**Iter**		**Nodes**	
		**Avg**	**Std**	**Avg**	**Std**	**Avg**	**Std**
[70,70]	[21]	6,518.2	869.2	19,358.8	926.4	9,586.3	749.3
	[19]	5,208.1	903.4	18,308.8	908.7	6,762.1	794.8
	Our	362.3	44.9	4,588.5	303.4	1,092.9	209.6
[70,100]	[21]	-	-	-	-	-	-
	[19]	6,691.2	923.6	23,650.8	936.2	9,834.3	792.8
	Our	528.3	65.6	5,186.3	291.5	1,394.1	287.1
[70,150]	[21]	-	-	-	-	-	-
	[19]	-	-	-	-	-	-
	Our	1,028.9	635.6	7,616.4	189.6	1,691.6	272.4
[100,150]	[21]	-	-	-	-	-	-
	[19]	-	-	-	-	-	-
	Our	1,124.4	603.8	7,096.3	193.1	1,702.2	243.4
[150,150]	[21]	-	-	-	-	-	-
	[19]	-	-	-	-	-	-
	Our	1,149.2	678.5	8,076.4	201.3	2,001.8	292.7
[150,200]	[21]	-	-	-	-	-	-
	[19]	-	-	-	-	-	-
	Our	2,048.5	728.9	9,806.7	416.8	2,671.9	397.5
[150,300]	[21]	-	-	-	-	-	-
	[19]	-	-	-	-	-	-
	Our	3,892.7	969.4	10,903.5	971.5	3,402.6	873.4
[200,300]	[21]	-	-	-	-	-	-
	[19]	-	-	-	-	-	-
	Our	3,912.8	917.2	9,938.3	911.7	3,521.5	816.3
[300,300]	[21]	-	-	-	-	-	-
	[19]	-	-	-	-	-	-
	Our	4,025.1	909.5	1,109.3	891.5	3,612.3	834.7
[300,400]	[21]	-	-	-	-	-	-
	[19]	-	-	-	-	-	-
	Our	4,875.5	962.5	14,946.1	938.6	5,827.5	972.8
[300,500]	[21]	-	-	-	-	-	-
	[19]	-	-	-	-	-	-
	Our	5,962.6	978.6	16,592.7	995.7	6,987.2	957.4

We first solve several sample examples, where Examples 1-3 and Examples 4-5 come from Ref. [28] and Ref. [29], respectively. The corresponding computational results are summarized in Table 1.

### Example 1

$$\begin{aligned}& \min \frac{-x_{1}+2x_{2}+2}{3x_{1}-4x_{2}+5}+\frac{4x_{1}-3x_{2}+4}{-2x _{1}+x_{2}+3} \\& {\text{s.t.}}\quad x_{1}+x_{2}\leq1.5, \quad\quad x_{1}\leq x_{2}, \quad\quad 0\leq x_{1} \leq1, \quad\quad 0\leq x_{2}\leq1. \end{aligned}$$

### Example 2

$$\begin{aligned}& \min \frac{-x_{1}+2x_{2}+2}{3x_{1}-4x_{2}+5}\times\frac{4x_{1}-3x_{2}+4}{-2x _{1}+x_{2}+3} \\& {\text{s.t.}}\quad x_{1}+x_{2}\leq1.5,\quad\quad x_{1}\leq x_{2}, \quad\quad 0\leq x_{1} \leq1, \quad\quad 0\leq x_{2}\leq1. \end{aligned}$$

### Example 3

$$\begin{aligned}& \min \prod_{i=1}^{6} \frac{\langle c^{i},x \rangle +r_{i}}{\langle d^{i},x\rangle +s_{i}} \\& {\text{s.t.}}\quad Ax\leq b,\quad\quad x\geq0, \end{aligned}$$

where

$$\begin{aligned}& {c}^{1}=( -0.2, -0.7, -0.1, 0.4, 0.0, 0.8, 0.1, -0.8, -0.2, 0.0, 0.1, 0.4), \quad\quad r _{1}= 21, \\& {d}^{1}=( 0.2, 0.5, -0.6, 0.1, 0.6, 0.4, -0.4, -0.3, 0.7, 0.5, 0.4, -0.1), \quad\quad s _{1}= 13.3, \\& {c}^{2}=( -0.1, 0.1, -0.4, -0.1, -0.1, 0.4, 0.2, 0.5, 0.3, -0.4, -0.3, 0.3), \quad\quad r _{2}= 16.3, \\& {d}^{2}=( -0.3, -0.2, -0.7, 0.1, 0.2, -0.2, -0.5, 0.4, 0.3, 0.0, 0.6, -0.5), \quad\quad s _{2}= 16, \\& {c}^{3}=( 0.8, 0.0, -0.1, 0.4, 0.2, 0.1, -0.5, 0.0, 0.5, 0.6, -0.3, -0.4), \quad\quad r _{3}= 3.7, \\& {d}^{3}=( 0.1, 0.0, 0.0, 0.3, 0.2, 0.7, 0.4, 0.2, -0.1, -0.5, 0.6, -0.1), \quad\quad s _{3}= 16.7, \\& {c}^{4}=( 0.6, 0.2, 0.2, -0.3, 0.5, 0.4, 0.1, 0.6, -0.3, 0.3, 0.4, 0.3), \quad\quad r _{4}= -1.8, \\& {d}^{4}=( -0.3, 0.0, 0.0, -0.5, -0.1, 0.2, 0.6, -0.6, 0.1, -0.2, 0.8, -0.3), \quad\quad s _{4}= 21.5, \\& {c}^{5}=( -0.3, -0.3, 0.5, 0.1, 0.2, -0.5, 0.1, 0.2, 0.0, 0.6, 0.3, -0.2), \quad\quad r _{5}= 5, \\& {d}^{5}=( 0.3, 0.0, 0.3, 0.0, -0.8, -0.3, 0.3, -0.9, -0.1, -0.6, -0.1, 0.2), \quad\quad s _{5}= 18.7, \\& {c}^{6}=( 0.2, -0.1, 0.0, 0.0, -0.2, -0.4, 0.0, -0.6, 0.8, -0.2, 0.0, -0.1), \quad\quad r _{6}= 12.7, \\& {d}^{6}=( 0.0, 0.6, 0.0, 0.1, 0.0, -0.2, 0.0, -0.5, 0.2, -0.3, 0.3, 0.1), \quad\quad s _{6}= 19.2, \\& A=\left [ \textstyle\begin{array}{@{}c@{\quad}c@{\quad}c@{\quad}c@{\quad}c@{\quad}c@{\quad}c@{\quad}c@{\quad}c@{\quad}c@{\quad}c@{\quad}c@{}} 1.9 & 0.0 & -0.2 & -1.5 & 1.8 & 0.9 & -1.0 & 4.5 & 4.5 & -3.5 & -1.8 & -4.8 \\ 2.9 & 3.7 & -4.8 & -1.9 & 1.8 & -3.7 & 1.8 & 2.5 & -2.9 & 1.9 & -3 & 3.2 \\ 3.3 & 2.4 & 3.3 & 4.8 & -0.3 & 3.9 & 0.8 & -1.7 & 2.0 & -0.3 & -1.8 & 2.2 \\ -4.3 & 1.8 & 2.1 & -4.5 & -0.5 & 2.4 & 1.4 & -0.3 & -2.0 & -2.8 & 0.4 & 4.5 \\ 1.5 & -0.3 & 0.4 & 1.2 & 1.1 & 1.9 & 1.5 & -1.2 & -3.3 & 4.4 & 3.2 & -4.3 \\ -3.2 & 2.4 & -4.5 & -1.0 & -2.7 & 3.7 & -0.1 & 3.9 & -1.9 & 3.2 & 2.1 & 1.3 \\ 0.9 & 0.5 & 4.0 & -1.5 & 1.2 & -1.5 & 1.2 & -3.7 & -0.1 & 0.0 & -2.4 & -4.1 \\ -4.1 & -4.5 & 2.2 & -3.1 & 4.4 & 4.8 & -3.4 & 2.2 & -2.1 & 2.3 & 2.6 & -1.4 \\ 2.4 & 2.3 & 4.7 & -1.7 & -1.6 & 3.8 & -4.0 & 1.3 & -0.4 & -0.4 & 2.9 & 1.2 \\ 0.0 & -3.2 & -0.2 & 2.0 & -2.9 & 2.7 & 3.1 & 2.9 & -2.6 & -4.3 & 0.2 & 4.6 \\ -1.3 & -0.9 & 3.4 & 3.9 & 4.9 & 2.3 & -3.0 & -1.5 & 2.5 & -1.7 & 1.7 & -2.9 \\ 3.5 & 3.4 & 2.5 & -0.4 & -4.5 & 2.8 & -1.7 & 2.1 & -2.9 & -4.7 & 1.3 & 4.5 \\ 1.9 & -0.9 & -3.3 & -2.3 & 1.6 & -0.5 & -4.9 & 3.0 & -4.9 & 3.6 & -3.7 & 2.2 \\ -1.4 & 3.5 & -2.8 & -1.2 & -4.7 & -3.2 & 2.2 & -4.0 & 2.8 & 3.3 & 4.4 & -3.1 \\ -2.1 & 2.6 & -3.9 & 1.0 & 2.3 & 1.8 & 4.2 & 1.8 & 2.7 & 0.9 & 3.3 & 1.7 \end{array}\displaystyle \right ] , \\& \begin{aligned} b&=(-20.1, -1.0, 82.6, 14.6, 37.7, 40.7, -23, \\ &\quad{} 47.4, 83.0, 9.9, 33.7, 49.1, 14.0, -45.6, 30.4)^{ \top}. \end{aligned} \end{aligned}$$

### Example 4

$$\begin{aligned}& \min \prod_{i=1}^{3}f_{i}(x) \\& {\text{s.t.}}\quad x_{1}+2x_{2}\leq10, \quad\quad 0\leq x \leq10, \quad\quad 0\leq x\leq4, \end{aligned}$$

where

$$\begin{aligned}& f_{1}(x)=(x_{1}-1)^{2}+(x_{2}-1)^{2}+1, \\& f_{2}(x)=(x_{1}-2)^{2}+(x_{2}-3)^{2}+1, \\& f_{3}(x)=(x_{1}-4)^{2}+(x_{2}-2)^{2}+1. \end{aligned}$$

### Example 5

$$\begin{aligned}& \min \prod_{i=1}^{3}f_{i}(x) \\& {\text{s.t.}}\quad (x_{1}-2)^{2}+(x_{2}-3)^{2} \leq1, \quad\quad 0\leq x\leq3, \quad\quad 0 \leq x\leq3, \end{aligned}$$

where

$$\begin{aligned}& f_{1}(x)=5x_{1}^{4}+x_{2}^{4}, \\& f_{2}(x)=3(x_{1}-5)^{4}+10(x_{2}-3)^{4}, \\& f_{3}(x)=7(x_{1}-2)^{4}+2(x_{2}-4)^{4}. \end{aligned}$$

Note that for solving Examples 4 and 5 we chose $(l_{1},l_{2},l_{3})=(1,1,1)$, $(u_{1},u_{2},u_{3})=(12,7,12)$ and $(l_{1},l_{2},l_{3})=(13,54,2)$, $(u_{1},u_{2},u_{3})=(450,850,105)$ which come from Ref. [29], respectively. In addition, we notice that the algorithm in [21] cannot be reasonable to solve Examples 4 and 5, and so we do not use it for solving them.

From Table 1, it can be seen easily that the proposed algorithm requires less computational time for solving Examples 1-5 compared with the ones in [19, 21] with the same $\varepsilon>0$ value. This is because the number of iterations and the maximal number of the interesting grid points restored are less than the ones in [19, 21] from Table 1, which means that the total number of the interesting grid points considered by the proposed algorithm is less than the one of the algorithms in [19, 21]. Also, in the three algorithms ([19, 21] and ours), notice that the main computational time is to check feasibility of linear programs at interesting grid points. Hence, the more interesting grid points are considered, the more computational time will be required.

Next, we apply the three algorithms ([19, 21] and our own) to randomly generated examples as follows.

$$\begin{aligned}& \min\prod_{i=1}^{p}c_{i}^{\top}x \\& \text{s.t.}\quad x\in X=\bigl\{ x\in\mathbb{R}^{n}:Ax\geq b, L\leq x \leq V\bigr\} , \end{aligned}$$

where all elements of $c_{i}\in\mathbb{R}^{n}$ and $L\in\mathbb{R} ^{n}$ are random numbers generated from the interval $[0,1]$; $b\in\mathbb{R}^{m}$, $V\in\mathbb{R}^{n}$ are randomly generated vectors with all components belonging to $(1,2)$; and each element of $A\in\mathbb{R}^{m\times n}$ is randomly generated in $[-1,1]$. Nineteen examples for selected combinations of *m* (number of constraints), *n* (number of variables), and *p* (number of linear functions in the objective function), altogether 190 randomly generated test instances are solved. The approximation error is fixed at $\varepsilon=0.01$, and the average computational results (standard deviation) are obtained by running the algorithms ([19, 21] and ours) for 10 times. Table 2 shows the numerical results for solving instances when $(m,n)=(50,50)$, *p* changed in $\{2,4,5,6,7,8,9,10\}$. Similarly, as $p=4$ and $(m,n)$ is changed, the computational results are listed in Table 3. In Tables 2 and 3, ‘-’ means the problem cannot be solved within two hours.

It can be seen from Tables 2 and 3 that the proposed algorithm needs fewer iterations and interesting grid points, and so requires less computational time for solving this kind of random problems, compared with the algorithms given by [19, 21]. Also, it is shown by Tables 2 and 3 that the performance of the algorithms is strongly affected by changes in *n* and *p*, specially, when *p* increases. The reason is that the number of operations required by the algorithms ([19, 21] and ours) is an exponential increase with *p* increasing according to the corresponding computational complexity results.

It is worth mentioning from Tables 2 and 3 that the computational time of the proposed algorithm increases with *n* and *p* increasing, but not as sharply as the algorithms in [19, 21]. For example, in Table 2, the instances cannot be solved by the algorithms in [19, 21] within two hours when $p\geq6$ and $p\geq7$, respectively, while the presented algorithm can solve all instances with *p* increasing 2 to 10 in less than two hours. This is due to the fact that the main computational cost of the algorithms ([19, 21] and ours) is the solution of linear feasibility problems at the interesting grid points. That is to say, the computational time for solving this kind of problems is directly affected by the number of interesting grid points. We notice that for the algorithms in [19, 21], the number of iterations and interesting grid points checked at each iteration increases with *p* increasing. However, for the proposed algorithm, *p* is related to the number of iterations (see Step (k2) in the proposed algorithm) and independent of the number of interesting grid points checked at each iteration (see Step (k1) in the proposed algorithm). This means that the proposed algorithm requires fewer interesting grid points considered and less computational time than the ones of the algorithms in [19, 21] for solving this kind of random problems. Moreover, from Table 3, notice that the algorithms in [19, 21] cannot solve the instances within two hours when $n\geq150$ and $p=4$, but all instances selected can be solved by the proposed algorithm within no more than two hours. This is mainly because the more interesting grid points are considered, the more the feasibility of linear programs with *n* variables should be checked. On the other hand, note that the interesting grid points considered by the proposed algorithm are much fewer than the ones considered by the algorithms [19, 21]. And so the increase of the computational time of the proposed algorithm is not as sharp as the algorithms [19, 21] with *n* increasing.

A comparison of the performance of the algorithms ([19, 21] and our own), the numerical results in Tables 1-3 show that the proposed algorithm is effective and the computational results can be obtained within a reasonable time.

## Results and discussion

In this work, a new solution algorithm for globally solving a class of generalized fractional programming problems is presented. As further work, we think the ideas can be extended to more general type optimization problems, in which each $c_{i}^{\top}x+c_{0i}$, $d_{i} ^{\top}x+d_{0i}$ in the objective function to problem (P) is replaced with a convex function, respectively.

## Conclusion

This article proposes a new approximation algorithm for solving a class of fractional programming problems (P) without the assumptions on quasi-concavity or low-rank. In order to solve this problem, the original problem (P) is first converted into a *p*-dimensional equivalent one with a box constrained set, we then give a new approximation algorithm which can be more easily implemented compared with the ones given in [19, 21]. Moreover, the computational complexity of such an algorithm can be derived to show that it is an FPTAS when *p* is fixed, and that its computational time is an exponential increase with *p* increasing. Also, the complexity results can be used as an indicator of the difficulty of some optimization problems falling into the category of (P), and so we should expect to design a more sophisticated approach where its performance is at least as good. Additionally, this article not only gives a provable bound on the running time of the proposed algorithm, but also guarantees the quality of the solution obtained to problem (P).
